# Exploring exosomes: novel diagnostic and therapeutic frontiers in thyroid cancer

**DOI:** 10.3389/fphar.2024.1431581

**Published:** 2024-11-08

**Authors:** Sicheng Zhang, Yan Yang, Dianri Wang, Xueting Yang, Yongcong Cai, Chunyan Shui, Ruoyi Yang, Wen Tian, Chao Li

**Affiliations:** ^1^ Department of Head and Neck Surgery, Sichuan Clinical Research Center for Cancer, Sichuan Cancer Hospital and Institute, Sichuan Cancer Center, Affiliated Cancer Hospital of University of Electronic Science and Technology of China, Chengdu, China; ^2^ Department of Oral and Maxillofacial Surgery, Guizhou Medical University, Guiyang, China; ^3^ Department of General Surgery, Chinese People’s Liberation Army General Hospital, Beijing, China

**Keywords:** thyroid cancer, exosome, tumorigenesis, diagnosis, therapy

## Abstract

In recent years, the incidence of thyroid cancer has surged globally, posing significant challenges in its diagnosis, treatment, and prognosis. Exosomes, as a class of extracellular vesicles, are secreted by nearly all cell types and encapsulate a variety of nucleic acids and proteins reflective of their cell of origin, thereby facilitating critical intercellular communication. Recent advancements in understanding these exosomes have catalyzed their application in oncology, particularly through uncovering their roles in the pathogenesis, diagnosis, and therapy of cancers. Notably, the latest literature highlights the integral role of exosomes in refining diagnostic techniques, enhancing targeted therapies, optimizing radiotherapy outcomes, and advancing immunotherapeutic approaches in thyroid cancer management. This review provides a current synthesis of the implications of exosomes in thyroid cancer tumorigenesis and progression, as well as their emerging applications in diagnosis and treatment strategies. Furthermore, we discuss the profound clinical potential of exosome-based interventions in managing thyroid cancer, serving as a foundational reference for future therapeutic developments.

## 1 Introduction

**FIGURE 1:**
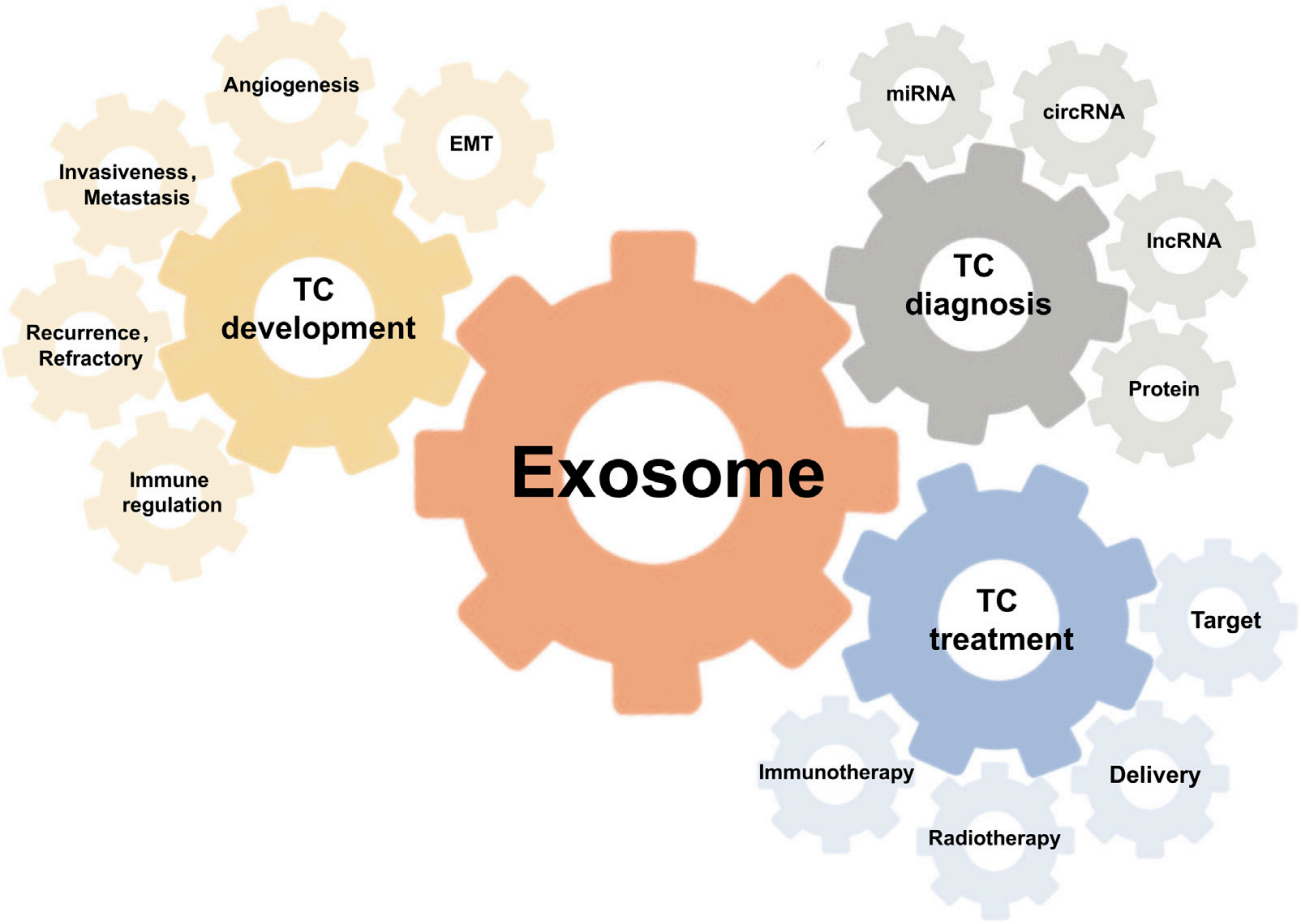
Illustration of the implications of exosomes in thyroid cancer.

In the past decade, thyroid cancer (TC) has emerged as the most prevalent endocrine malignancy, accounting for approximately 3.8% of all newly diagnosed cancers worldwide (Pavlidis et al., 2021). This alarming rise underscores the critical need for innovative management strategies to enhance patient outcomes. Exosomes, nano-sized extracellular vesicles secreted by various cell types, play a fundamental role in intercellular communication by transmitting bioactive molecules, thereby reflecting the molecular signature of their cells of origin. Extensive studies across multiple cancer types, including breast cancer (da Fonseca Alves et al., 2024), lung cancer (Li M. Y. et al., 2021), liver cancer (Wang H. et al., 2019), colorectal cancer (Chen C. et al., 2022), prostate cancer (Lorenc et al., 2020), and pancreatic cancer (Fang et al., 2023), have elucidated the pivotal role of exosomes in tumorigenesis and disease progression. Moreover, recent advancements have demonstrated the significant potential of exosome-based clinical applications, including cancer diagnosis (Zhong et al., 2024), monitoring (Li S. et al., 2023), and therapeutic interventions (Yue et al., 2023; Si et al., 2024). Specifically in thyroid cancer, studies have demonstrated that exosomes derived from tumor cells facilitate both the progression and the metastasis (Feng et al., 2020). Further exploration of exosome-mediated mechanisms may reveal novel insights into thyroid cancer pathophysiology (Wang et al., 2024). Additionally, the non-invasive nature and high specificity of exosome-based diagnostic and therapeutic strategies hold promise for revolutionizing the management of thyroid cancer, potentially ushering in a new era of precision medicine (Figure 1).

## 2 Thyroid cancer

### 2.1 Clinical classification

Differentiated thyroid cancer (DTC), which originates from thyroid follicular epithelial cells, accounts for over 95% of all cases (Uludağ et al., 2018). This category primarily includes papillary thyroid cancer (PTC) and follicular thyroid cancer (FTC). PTC, the most prevalent subtype, is characterized by its indolent behavior, favorable prognosis, and a propensity for regional lymphatic spread, particularly to the cervical lymph nodes, with less frequent metastasis to distant sites such as the lungs and bones (Schlumberger and Leboulleux, 2021). In contrast, poorly differentiated thyroid cancer (PDTC) and the Hürthle cell variant, which are categorized as high-risk, generally present with poorer outcomes (Cabanillas et al., 2016; Houten et al., 2022). Anaplastic thyroid cancer (ATC), although constituting less than 1% of all thyroid cancers, is the most lethal form, exhibiting an extremely aggressive nature and rapid disease progression. ATC commonly infiltrates adjacent neck structures and metastasizes to distant organs, leading to a median survival of merely 3 months (Lin et al., 2019). Medullary thyroid carcinoma (MTC), which accounts for 1%–2% of all thyroid cancers, originates from parafollicular C cells and is distinguished by its high metastatic potential and poor prognosis, often identified through elevated serum calcitonin levels (Okafor et al., 2021; Kaliszewski et al., 2022).

### 2.2 Clinical diagnosis

**FIGURE 2:**
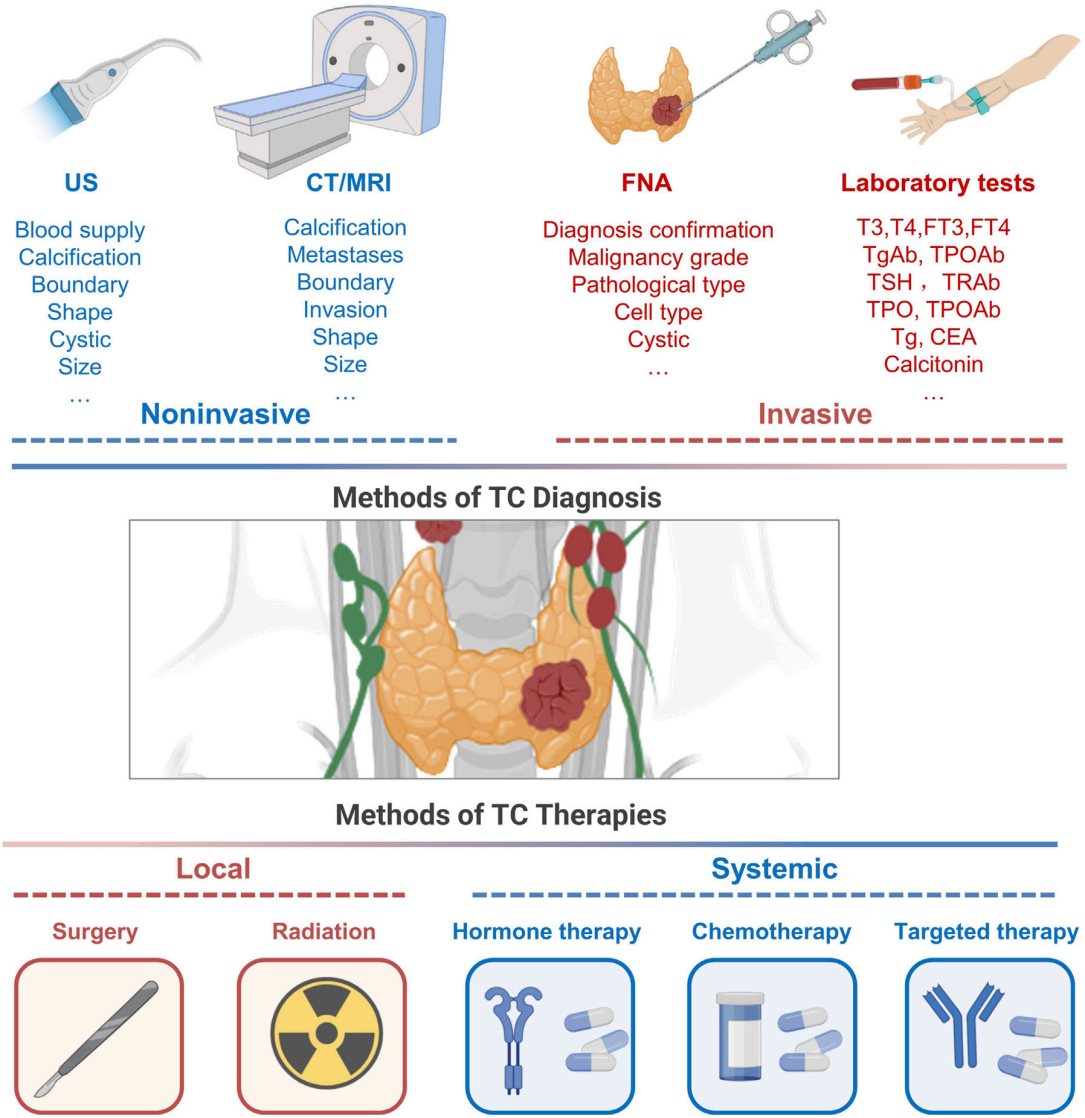
Conventional approaches to thyroid cancer diagnosis and management.

The primary diagnostic modalities for DTC include ultrasound and fine-needle aspiration (FNA), which are further supported by cytological evaluations (Haugen et al., 2016; Filetti et al., 2019). Additionally, biochemical assays and genetic screenings, such as those detecting BRAF and TERT mutations, play a crucial role in refining diagnosis and prognostication (Houten et al., 2022). ATC, known for its rapid progression, requires swift histopathological evaluation, frequently complemented by immunohistochemistry to confirm diagnoses. The diagnostic approach for MTC integrates imaging modalities, serum biochemical markers, and genetic testing, highlighting the pivotal role of early and accurate detection in enhancing patient outcomes (Wells Jr et al., 2015; Filetti et al., 2019) (Figure 2). Given the non-invasive and convenient nature of peripheral blood assays, they are increasingly recognized as viable options for continuous monitoring of thyroid cancer. Consequently, the investigation of biomarkers in peripheral blood, particularly exosomal markers, is gaining prominence, reflecting their crucial role in both diagnosing TC and monitoring patient progress.

### 2.3 Clinical management

The clinical management of DTC typically involves a combination of surgery, radioactive iodine (RAI) therapy, and TSH suppression therapy to minimize the risk of recurrence (Haugen et al., 2016). In contrast, ATC and MTC, known for their rapid progression and high recurrence rates, necessitate more aggressive treatment strategies. These include extensive surgery, radiation, systemic therapies, and the incorporation of emerging modalities such as immunotherapy (Dierks et al., 2021). Given the resistance and recurrence of ATC and MTC, identifying innovative therapeutic strategies is crucial for improving survival. Exosomes, extracellular vesicles from cells, are central in tumorigenesis, progression, and resistance within the tumor microenvironment. Their role in these processes marks them as key targets for novel therapies, especially as vehicles for targeted drug delivery. In the following sections, we will explore these aspects of exosome function and their therapeutic potential in detail.

Diagnostic procedures for TC encompass a range of modalities, including ultrasonography, CT/MRI imaging, FNA, and circulating blood tests. Traditional approaches to managing TC predominantly involve a combination of surgical intervention, localized radiation using RAI, therapeutic strategies to suppress TSH, chemotherapy, and targeted therapy. US, ultrasonography; FNA, fine-needle aspiration; T3, triiodothyronine; T4, thyroxine; Tg, thyroglobulin; TPO, thyroid peroxidase; TSH, thyroid stimulating hormone; CEA, carcinoembryonic antigen; RAI, radio-active iodine.

## 3 Exosomes

**FIGURE 3:**
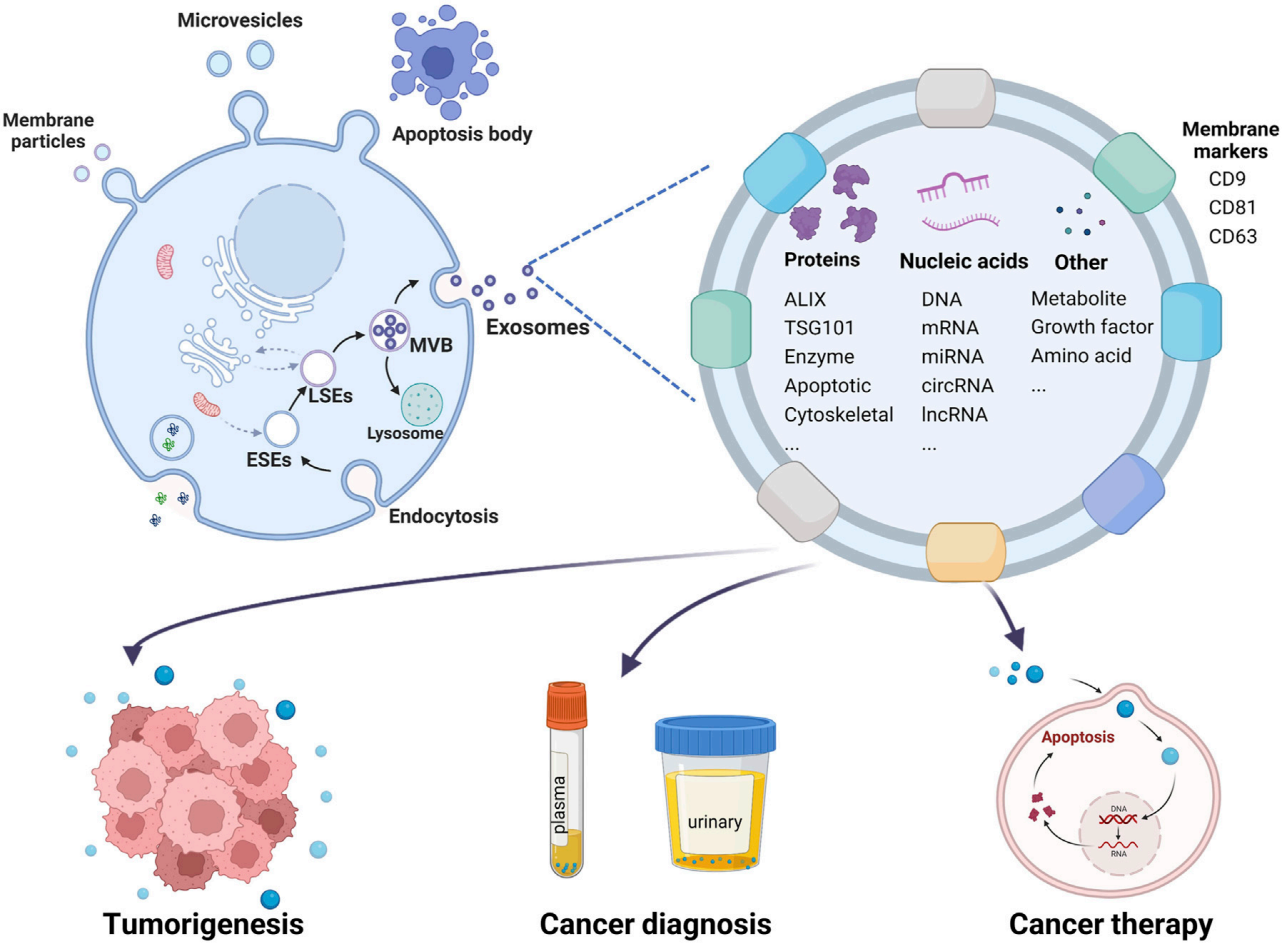
Schematic illustration of exosome biogenesis, identification, and roles in tumorigenesis, diagnosis, and therapy. This diagram differentiates various types of extracellular vesicles by size and secretion pathways: membrane particles (50–600 nm), microvesicles (100–1,000 nm) originating from the cell surface, and apoptotic bodies (1,000–5,000 nm) from apoptotic cells. Exosomes, derived from multivesicular bodies (MVBs), are depicted as nanoparticles with an average diameter of 40–150 nm. They contain nucleic acids, proteins, lipids, and metabolites, playing crucial roles in intercellular communication. Exosomes influence the tumor microenvironment, aid in tumor formation, and are utilized in blood or urine for early tumor diagnosis. Additionally, they serve as vehicles for targeted cancer therapy.

Over the past four decades, since their initial identification by Pan and Johnstone (1983), Johnstone et al., (1987), exosomes have been recognized to originate from a diverse array of cell types including reticulocytes (Pan and Johnstone, 1983), immunocytes (Li et al., 2019), neurons (Jiang D. et al., 2020) and tumor cells (Hardin et al., 2018; Dai D. et al., 2020; Wang Y. et al., 2020). These extracellular vesicles can be isolated from various bodily fluids such as serum (Dai W. et al., 2020; Rao et al., 2020), urine (Huang et al., 2020), saliva (Nik Mohamed Kamal et al., 2020), semen (Su et al., 2021), and cerebrospinal fluid (Jia et al., 2019). Characterized by their distinctive cup-like morphology when visualized through transmission electron microscopy, exosomes are nanoparticles typically ranging in diameter from 40 to 150 nm (Zhang et al., 2020; Wang D. et al., 2022). Their identification can be confirmed through Western blot analysis using specific protein markers, such as CD9, CD81, CD63, TSG101, and Alix (Kalluri and LeBleu, 2020). As a subclass of the larger extracellular vesicle family, exosomes are distinguished from other types, including microvesicles, microparticles, and apoptotic bodies, based on their size, biogenesis, and molecular composition (Zhang et al., 2021) (Figure 3).

### 3.1 Exosome biogenesis

Exosome biogenesis is initiated through the formation of early-sorting endosomes (ESEs), which arise via endocytic uptake of extracellular substances and involve contributions from the Golgi apparatus and endoplasmic reticulum. These ESEs mature into late-sorting endosomes (LSEs) and subsequently develop into multivesicular bodies (MVBs) containing intraluminal vesicles (ILVs) (Minciacchi et al., 2015; Al-Sowayan et al., 2020). During the maturation process of MVBs, specific proteins, nucleic acids, and lipids are selectively incorporated into ILVs, which are then released into the extracellular space as exosomes upon fusion of MVBs with the plasma membrane. Alternatively, MVBs may be targeted for degradation through lysosomal pathways or recycled back to the plasma membrane, depending on cellular requirements (Figure 3).

### 3.2 Exosome structure and contents

Initially considered mere cellular waste, exosomes have since been recognized for their crucial role in cell-to-cell communication (Harding and Stahl, 1983; Zhang et al., 2019). These phospholipid bilayer-enclosed nanoparticles contain a diverse array of biomolecules, including proteins, nucleic acids, RNAs, DNAs, amino acids, and metabolites, all of which can elicit specific regulatory effects on recipient cells (Zhang et al., 2016). Numerous proteins involved in exosome biogenesis also serve as markers for these vesicles (Kalluri and LeBleu, 2020). Recent research has further elucidated the critical role of exosomes in tumorigenesis and their potential to refine diagnostic and therapeutic strategies in oncology (Xu et al., 2020; Glass and Coffey, 2022; Zhou et al., 2022) (Figure 3).

### 3.3 Exosome isolation

Exosome isolation utilizes a variety of techniques, including ultracentrifugation, immunoaffinity capture, ultrafiltration, and polymer-based precipitation, however, no single method can simultaneously achieve high yield and purity (Yang X. X. et al., 2019). Ultracentrifugation, a conventional method involving prolonged spinning at 100,000 g, is often contaminated and limited in throughput (Herroon et al., 2019). Alternative methods, including polymer-based precipitation and density-gradient ultracentrifugation, face similar challenges in yield and purity (Gardiner et al., 2016; Konoshenko et al., 2018). Despite the availability of alternative methods like polymer-based precipitation, density-gradient ultracentrifugation, and immunoprecipitation, these extraction techniques continue to grapple with diminishing yields and purity challenges. Moreover, distinguishing exosomes from other extracellular vesicle proves challenging due to overlapping properties (Cocozza et al., 2020). Therefore, in this review, we use the term “exosome” interchangeably with “extracellular vesicles” as per International Society for Extracellular Vesicles (ISEV) guidelines (Théry et al., 2018).

## 4 Exosomes in TC development

**FIGURE 4:**
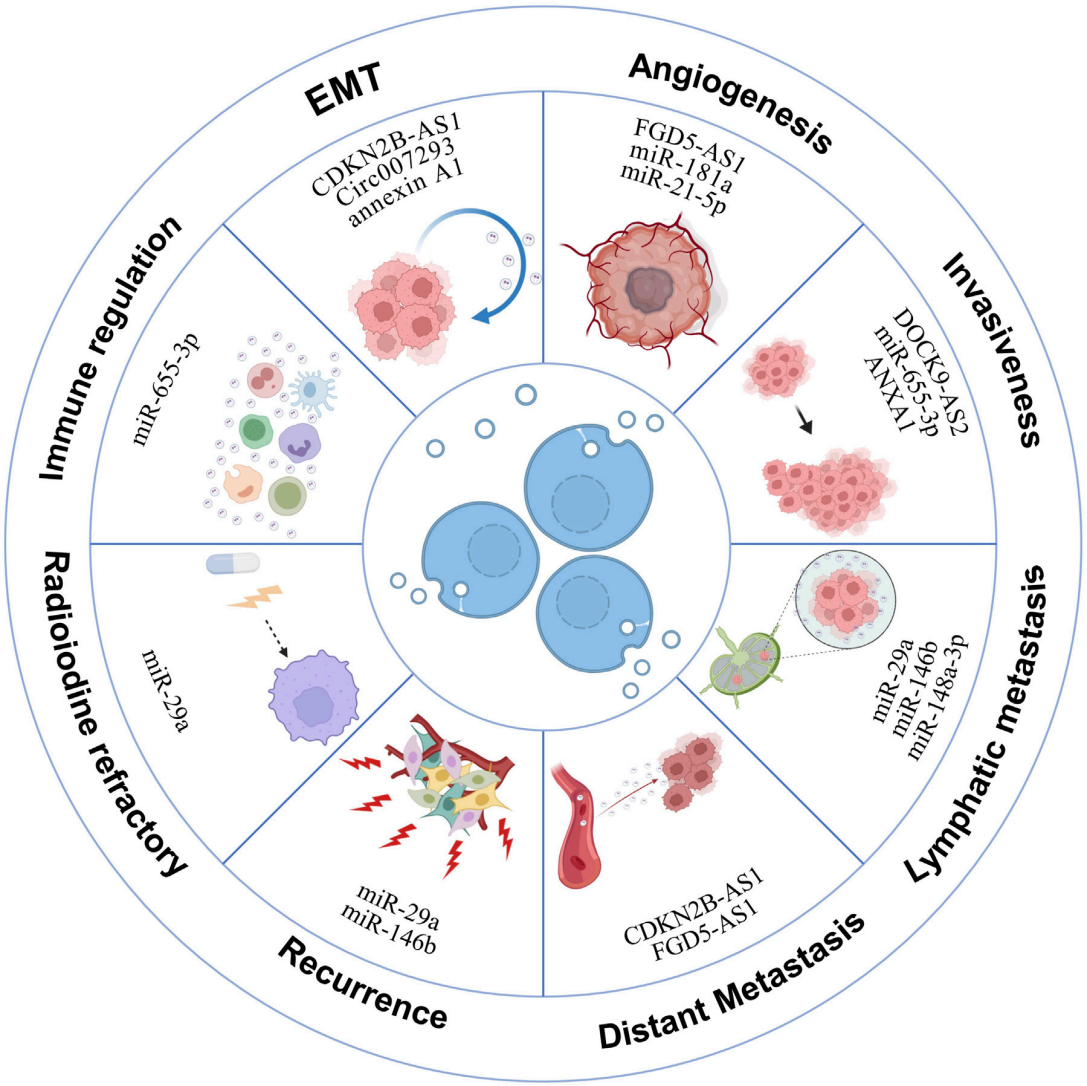
Exosomal mechanisms in thyroid cancer development. This illustration delineates how exosomes derived from thyroid cancer cells promote various oncogenic processes. These include orchestrating angiogenesis, facilitating the epithelial-to-mesenchymal transition, and contributing to tumor growth, invasion, and metastasis. Additionally, exosomes play roles in immunosuppression and in the development of resistance to iodine-131 radioiodine treatment.

Recent research has increasingly focused on the role of exosomes in modulating physiological and pathological processes in cancer, including thyroid cancer (Zhang and Yu, 2019). In TC patients, a significant presence of exosomes in tissues and blood indicates their critical role in carcinogenesis. Originating from thyroid cancer cells, these exosomes facilitate tumor development, progression, metastasis, and radioiodine resistance by transferring their contents to other cells (Li Q. et al., 2021) (Figure 4). Broadly, the exosomal mechanisms underlying TC development encompass several key components, as illustrated in Table 1. Research into exosomes in thyroid cancer development is poised to facilitate the identification of novel therapeutic targets, thereby offering promising avenues for more effective treatments.

**TABLE 1:** The role of exosomes in thyroid cancer tumorigenesis.

Exosome origin	Exosomal contents	Functions	Reference
Epithelial-mesenchymal transition			
Cancer stem-like cells	CDKN2B-AS1	Regulate EMT and TGFβ/Smad signaling	Wu et al. (2022)
PTC patient serum and PTC cell culture medium	circ007293	Induce EMT and augment the invasive and migratory abilities of PTC cells	Lin et al. (2021)
Thyroid cancer cell line (SW579)	annexin A1	Promote EMT, proliferation and invasion of the thyroid follicular epithelial cell line	Li Q. et al. (2021)
Serum from PTC patients with LNM	SRC, TLN1, ITGB2 and CAPNS1	Associated with EMT in TC patients with lymph node metastasis	Luo et al. (2018)
Cancer stem-like cells	LncRNA, MALAT1, SLUG, SOX2 and linc-ROR	Induce EMT and inculcate local tumor microenvironment and distant metastasis	Hardin et al. (2018)
Angiogenesis			
Thyroid cancer cells	FGD5-AS1	Promote angiogenesis, vascular permeability	Liu et al. (2022)
Hypoxic human PTC cell lines BCPAP	miR-181a	Induced angiogenesis and tumor growth *in vivo*	Wang et al. (2021)
Hypoxic PTC cells	miR-21-5p	Increase endothelial tube formation	Wu et al. (2019)
Invasiveness			
ATC cell line (8305C)	ANXA1	FC = 3.4 (vs. FTC) and FC = 3.5 (vs. Nthy-ori 3–1 cells)	Surman et al. (2024)
PTC patients	EGFR	High EGFR protein in PTC is disseminated through exosomes to the extracellular milieu	Al-Abdallah et al. (2023)
TC-CSCs	CDKN2B-AS1	Promote the malignant progression	Wu et al. (2023)
PTC patient serum and PTC cell culture medium	miR-655-3p	Inhibit PTC growth, and invasion	Qiao et al. (2022)
PTC cell lines (KTC-1, TPC-1)	DIO3OS	DIO3OS hindered the proliferation, invasion, and migration of PTC cells	Wang Y. et al. (2022)
Thyroid cancer cell line (SW579)	ANXA1	Promote the proliferation and invasion of the thyroid follicular epithelial cells	Li Q. et al. (2021)
PTC cell culture medium	circ007293	Augment the invasive and migratory abilities of PTC cells	Lin et al. (2021)
PTC-CSCs	lncRNA DOCK9-AS2	Aggravate the migration and invasion ability of PTC cells	Dai W. et al. (2020)
PTC cell lines	miR-146b-5p, miR-222-3p	Enhance migration and invasion ability of PTC cells *in vitro*	Jiang K. et al. (2020)
PTC cell lines	miRNA423-5p	Increase PTC cell migration and invasion	Ye et al. (2019)
Lymph node metastasis			
Serum from PTC patients	circTACC2, circBIRC6	Correlated with tumor LNM in PTC	Peng et al. (2024)
Plasma from PTC patients	miR-146b-5p, 222-3p	Correlated with tumor LNM	Jiang K. et al. (2020)
Serum from PTC patients	miR-485-3p	Correlated with tumor LNM in PTC	Dai D. et al. (2020)
Distant metastasis			
Patient plasma and cell lines	miR-519e-5p	Transferred to recipient CD8^+^ T cells and aid in tumor immune escape	Li G. et al. (2023)
CSC-like cells	CDKN2B-AS1	Drive TC distant metastasis	Wu et al. (2022)
TC cell lines	FGD5-AS1	Induce distant metastasis niche	Liu et al. (2022)
Recurrence and RAIR			
Serum from PTC patients	miR-29a	Patients with lower serum exosomal miR-29a may have a higher risk of recurrence	Wen et al. (2021)
PTC cell lines	miR-146b and miR-222	Potential biomarkers of PTC recurrence	Lee et al. (2015)
RAIR cell lines and plasma	miR-1296-5p	Involved in the pathogenesis of RAIR PTC by directly targeting Na+/I-symporter	Li et al. (2022)
Immune regulation			
PTC cell lines	ANXA1	Induce macrophage M2 polarization	Liu (2024)
PTC cell lines	miR-655-3p	Inhibit macrophage M2 polarization	Qiao et al. (2022)

ATC, anaplastic thyroid carcinoma; PTC, papillary thyroid cancer; FTC, follicular thyroid cancer; FC, fold change.

### 4.1 Epithelial-mesenchymal transition (EMT)

The epithelial-mesenchymal transition (EMT) plays a critical role in cancer initiation and progression, driving the phenotypic transformation of epithelial cells along the epithelial-mesenchymal spectrum (Zhang and Weinberg, 2018). TC cells release various cytokines into the tumor microenvironment (TME), inducing EMT in thyroid follicular epithelial cells and promoting extrathyroidal extension and distant metastasis (Puppin et al., 2008; Ma et al., 2014; Sponziello et al., 2016; Jarboe et al., 2021). Recent studies highlight exosomes and their cargoes as key mediators in this process, with potential as therapeutic targets to inhibit TC progression by regulating EMT (Kim et al., 2020). Lin et al. discovered that exosomal circ007293 triggers EMT and enhances invasiveness in PTC cells via the miR-653-5p/PAX6 axis (Lin et al., 2021). Similarly, Luo and colleagues linked exosomal proteins like SRC, TLN1, ITGB2, and CAPNS1 to EMT in TC patients with lymph node metastasis (LNM) (Luo et al., 2018). Furthermore, exosomal annexin A1 has been shown to stimulate EMT, proliferation, and invasion, contributing to the malignant transformation of thyroid cells (Li Q. et al., 2021). Additionally, cancer stem-like cells (CSCs), known for their self-renewal and asymmetric division capabilities, have been implicated in tumorigenesis and metastasis (Tang et al., 2016; Babaei et al., 2021). Recently, Wu and colleagues demonstrated that exosomal CDKN2B-AS1 from CSCs influences EMT and TGFβ/Smad signaling in human PTC and ATC cell lines, thereby promoting TC growth and metastasis (Wu et al., 2022). Hardin et al. further revealed that CSC-derived exosomes could induce EMT in non-cancerous thyroid cells via the transfer of linc-ROR, offering insights into potential therapeutic strategies targeting CSCs and their exosomes to combat aggressive TC (Hardin et al., 2018). These findings underscore the promise of exosome-based therapies in mitigating EMT-related alterations and reducing TC invasion and metastasis.

### 4.2 Angiogenesis

Angiogenesis plays a critical role in oncogenesis by expanding blood vessels that supply nutrients and remove wastes, thus facilitating malignant tumor growth (Rajabi et al., 2019). Studies have shown that exosomes secreted by cancer cells can enhance angiogenesis, supporting the progression of various tumors (Kosaka et al., 2013; Hsu et al., 2017; Du et al., 2020; Shang et al., 2020). Specifically, in thyroid cancer, angiogenesis is crucial for tumor progression, with DTC tissues exhibiting higher vascular densities compared to adjacent non-tumor tissues (Akslen and Livolsi, 2000; Ramsden, 2000; Rajabi et al., 2019). Research by Wu et al. highlighted increased exosomal miR-21-5p levels in the blood of PTC patients, which enhances angiogenesis in human umbilical vein endothelial cells (HUVECs) through the miR-21-5p/TGFβ and miR-21-5p/COL4α pathways under hypoxic conditions (Wu et al., 2019). Likewise, Wang’s research demonstrated that under hypoxia, exosomal miR-181a from PTC cells significantly increases cell proliferation, migration, and angiogenesis in HUVECs, ultimately leading to enhanced angiogenesis and tumor growth in nude mice (Wang et al., 2021). Liu et al. further reported that exosomal FGD5-AS1 influences angiogenesis and vascular permeability by modulating the miR-6838-5p/VAV2 axis in endothelial cells, pushing forward TC development and providing a new target for diagnosis and therapy (Liu et al., 2022). These findings suggest that targeting exosome-mediated angiogenesis might offer effective therapeutic strategies for thyroid cancer.

### 4.3 Invasiveness and metastasis

Over time, tumor cells gain the ability to infiltrate and interact with neighboring tissues, culminating in metastasis (Hardin et al., 2018). Hoshino et al. demonstrated that exosomes from tumors are internalized by organ-specific cells, playing a crucial role in metastasis (Hoshino et al., 2015). Recently, the promotion of TC invasiveness and metastasis by exosomes has been increasingly documented (Feng et al., 2020; Wang Y. et al., 2020).

Studies have demonstrated that the expression level of ANXA1 in exosomes derived from 8305C cells is 3.4 times higher than in exosomes from FTC cells and 3.5 times higher than in exosomes from normal cells (Surman et al., 2024). Moreover, elevated levels of ANXA1 in ATC-derived exosomes have been shown to promote the proliferation and invasion of thyroid follicular epithelial cells, contributing to their malignant transformation (Li Q. et al., 2021). Similarly, exosomal EGFR in PTC is linked to increased tumor aggressiveness (Al-Abdallah et al., 2023), and CSC-derived exosomes containing lncRNA DOCK9-AS2 and CDKN2B-AS1 are implicated in advancing thyroid cancer malignancy (Dai W. et al., 2020; Wu et al., 2023). Functional assays reveal that overexpression of exosomal miRNA423-5p increases PTC cell migration and invasion, while its silencing reduces these processes (Ye et al., 2019). Jiang’s research further identifies miR-146b-5p and miR-222-3p in exosomes as enhancers of PTC cell motility (Jiang K. et al., 2020), whereas Lin et al. found that exosomal circ007293 promotes invasiveness via the miR-653-5p/PAX6 axis (Lin et al., 2021). Conversely, exosomal DIO3OS and miR-655-3p were found to inhibit PTC cell proliferation and motility (Qiao et al., 2022; Wang Y. et al., 2022).

Lymph node metastasis (LNM) is a critical marker of malignancy severity in TC, associated with higher recurrence rates and worse outcomes (Nishino and Jacob, 2020). Exosomes play a key role in lymphangiogenesis and lymphatic network restructuring, facilitating LNM (Sun et al., 2019; Wang S. H. et al., 2019; Zhou et al., 2019). Specific exosomal miRNAs correlate with tumor size and LNM occurrence (Dai D. et al., 2020), and miR-6774-3p and miR-6879-5p serve as potential biomarkers for LNM diagnosis in PTC (Chen W. et al., 2022). Furthermore, Jiang’s investigation encompassed 49 PTC patients with LNM and 15 without LNM, revealing upregulated expression of exosomal miR-222-3p and miR-146b-5p in PTC patients with LNM (Jiang K. et al., 2020). Regarding exosome-derived proteins, Luo et al. postulated that integrin-associated proteins within serum-purified exosomes from PTC patients may facilitate the creation of premetastatic niches and contribute to subsequent LNM (Luo et al., 2018). These findings collectively underscore the significant role of exosomes in mediating the process of LNM in TC.

For ATC, distant metastasis to the lung or brain is common (Abe and Lam, 2021), with exosomes facilitating intercellular communication crucial for poor prognosis. Wu et al. examined the impact of CSCs-derived exosomal CDKN2B-AS1 on TC, utilizing mouse models to establish tumor formation and metastasis scenarios. Notably, observation of metastasis loci in the lung underscored the significant role of exosomal CDKN2B-AS1 in driving TC distant metastasis (Wu et al., 2022). Similarly, Liu et al. reported that exosomal lncRNAs like FGD5-AS1 induce tumor angiogenesis and support both local and distant metastatic niches (Liu et al., 2022). Additionally, research demonstrated that miR-519e-5p, harbored within exosomes from PTC, is transferred to recipient CD8^+^ T cells and aid in tumor immune escape in distant organs (Li G. et al., 2023).

These insights underline the complex role of exosomes in TC progression and metastasis, highlighting their potential as targets for therapeutic intervention.

### 4.4 Recurrence and radioiodine refractory

While the prognosis for most PTC patients is favorable, recurrence, especially in advanced stages and aggressive phenotypes, remains a challenge (Lundgren et al., 2006; Aragon Han et al., 2015). Exosomes serve as a valuable model for studying tumor recurrence and predicting associated risks (Soeda et al., 2019; Zhu et al., 2021). Regarding exosome-related TC recurrence, Wen et al. reported that among patients with low serum exosomal miR-29a levels, 25 out of 65 experienced PTC recurrence, indicating a higher risk associated with lower miR-29a levels (Wen et al., 2021). Additionally, Lee and colleagues identified overexpression of miR-146b and miR-222 in PTC cell-derived exosomes, proposing them as potential biomarkers for recurrence (Lee et al., 2015).

In addition to TC recurrence, another critical consideration is the emergence of RAIR cases. A subset of cases displays progressive behaviors, ultimately leading to the development of RAIR PTC. Remarkably, Li et al. have documented that the presence of circulating exosomal miR-1296-5p might play a contributory role in the pathogenesis of RAIR PTC by specifically targeting the Na^+^/I^−^ symporter (Li et al., 2022).

### 4.5 Immune regulation of TC

Tumor-derived exosomes can significantly impair anti-tumor immunity, thereby promoting tumor progression. For example, cancer cell-released exosomes have been shown to inhibit the function of NK cells (Liu et al., 2006) and stimulate tumor-promoting responses via interactions with B cells (Pucci et al., 2016). They also facilitate immune evasion by impairing T cell function through exosome-mediated PD-L1 expression (Kim et al., 2019) and promote the expansion of myeloid-derived suppressor cells at both primary and metastatic sites (Chalmin et al., 2010). Among cancers, TC is notably immunogenic (Zhu et al., 2018; Chakladar et al., 2019). Zhu et al. found that stimulating NK cells with IL-15 increases exosome production, enhancing their potential for immunotherapy in various cancers, including thyroid cancer (Zhu et al., 2018). Additionally, Liu demonstrated that miR-374a/b-5p inhibits the growth of PTC cells by blocking the exosome-induced M2 macrophage polarization mediated by ANXA1 (Liu, 2024). Similarly, Qiao et al. showed that exosomal miR-655-3p suppresses cell growth, invasion, and M2 macrophage polarization in PTC by targeting CXCR4 (Qiao et al., 2022). These findings underscore the potential of leveraging exosomes as a novel delivery platform to advance immunotherapy for thyroid carcinoma, highlighting ongoing research efforts to harness this capability.

## 5 The role of exosomes in TC diagnosis

The primary diagnostic methods for TC include imaging and serum TSH measurement, followed by ultrasound-guided FNA, which, despite its invasiveness, is limited to diagnosis without aiding in therapy monitoring. Additionally, neck ultrasound for LNM detection reveals only half of the nodes identified during surgery. Although tissue biopsies offer high diagnostic accuracy, their invasiveness constrains their further use. Liquid biopsy, incorporating circulating tumor cells (CTCs), tumor DNA (ctDNA), cell-free DNA (cfDNA), and RNA (cfRNA), provides a non-invasive alternative for both diagnosing and monitoring cancer (Khatami and Tavangar, 2018; Rappa et al., 2019). Recent studies underscore the significant potential of exosomes as fluid biopsy tools in various cancers (Hesari et al., 2018; Huang et al., 2019; Yang et al., 2019a). For TC, experimental evidence also increasingly underscores the potential of exosomes as effective fluid biopsy tools (Table 2). The bilayer protection of circulating exosomes offers substantial advantages over CTCs and ctDNA in stability and integrity in the bloodstream (Junqueira-Neto et al., 2019). Furthermore, exosomal microRNAs, circular RNAs, and long non-coding RNAs are increasingly recognized for their critical roles in diagnosing TC, enhancing the accuracy and non-invasiveness of current diagnostic approaches.

**TABLE 2:** Prospective exosomal biomarkers of thyroid cancer.

Sample	Patients	Biomarker	Functions	Reference
miRNA				
Plasma	48 PTC M0,21 PTC M1	miR-519e-5p	Biomarker (Up), metastatic	Li G. et al. (2023)
Plasma	48 non-RAIR, 21 RAIR	miR-1296-5p	Biomarker (Up), RAIR	Li et al. (2022)
Serum	4 PTC, 4 NG	hsa-miR-129–2, 889	Biomarker (Down)	Xin et al. (2022)
Serum	PTC 56 -N1,58-N0	miR24-3p, 181a-5p, 146a-5p, 382-5p	Biomarker (Down)	Capriglione et al. (2022)
Plasma	PTC 34 -N1, 34-N0	miR-6774-3p, 6879-5p	Biomarker (Up)	Chen W. et al. (2022)
Plasma	17 PTC, 19 MNG	miR-146b-5p, 21a-5p	Biomarker (Up)	Delcorte et al. (2022b)
Serum	119 PTC, 100 HC	miR-29a	Biomarker (Down)	Wen et al. (2021)
Plasma	30 FTC, 30 FA	miR Let-7	Biomarker (Up)	Zabegina et al. (2020)
Plasma	PTC 49-N1, 15-N0	miR-146b-5p, 222-3p	Biomarker (Up), LNM	Jiang K. et al. (2020)
Plasma	51 PTC, 38 MNG	miR-16-2-3p, 223-5p	Biomarker (Up)	Liang et al. (2020)
Plasma	13 PTC, 7 MNG	miR-5189-3p	Biomarker (Up)	Pan et al. (2020)
Plasma	136 PTC, 92 MNG	miR-485-3p, 4433a-5p	Biomarker (Up), Risk	Dai D. et al. (2020)
Plasma	25 PTC, 25 HC	miR-346, 10a-5p, 34a-5p	Biomarker (Up)	Wang Z. et al. (2019)
Serum	60 PTC, 60 HC	miRNA423-5p	Biomarker (Up)	Ye et al. (2019)
Plasma	60 PTC, FTC, BN	miR-21-5p, 181a	Biomarker (Up)	Samsonov et al. (2016)
circRNA				
Serum	19 PTC, 18 HC	circTACC2, circBIRC6	Biomarker (Up), LNM	Peng et al. (2024)
Serum	164 PTC, 68 HC, 60 BN	hsa_circ_0082002, hsa_circ_0003863	Biomarker (Up), metastatic	Dai et al. (2023)
Serum	3 PTC, 3 MNG	circ-007293, 031752, 020135	Biomarker (Up)	Yang et al. (2019b)
lncRNA				
Blood	28 TC, 28 HC	lncRNA FGD5-AS1	Angiogenesis, Progression	Liu et al. (2022)
Plasma	54 PTC, 44 HC	lncRNA DOCK9-AS2	PTC progression	Dai W. et al. (2020)
Proteins				
Urinary	21 PTC	TIMP, Angiotensin-1	LNM	Wang C. Y. et al. (2022)
Urinary	16 PTC, FTC	Thyroglobulin	LNM, Stage	Huang et al. (2020)
Plasma	43 PTC, 43 HC	PD-L1	Stage	Wang G. et al. (2020)
Plasma	13 PTC, 18 MNG	Hsp27, Hsp60, and Hsp90	Biomarker (Up)	Caruso Bavisotto et al. (2019)
Serum	PTC 16-N1, 17-N0	SRC, TLN1, ITGB2, CAPNS1	Invasion, Metastasis	Luo et al. (2018)

RAIR, iodine-131 radioiodine refractory; LNM, lymph node metastasis; miRNA, microRNA; circRNA, circular RNA; lncRNA, Long noncoding RNA; PTC, papillary thyroid cancer; FTC, follicular thyroid cancer; FA, follicular adenomas; MNG, multinodular goiter; NG, nodular goiter; BN, Benign nodules; HC, Healthy control.

### 5.1 Exosomal miRNA

Exosomes are increasingly recognized for their stability as RNA carriers, offering a more reliable biomarker source than circulating plasma RNAs for disease diagnosis. They maintain RNA integrity even when stored at −20°C, proving essential for long-term studies (Sanz-Rubio et al., 2018). Recent advances show that exosomal miRNAs are potent diagnostic markers for TC, particularly PTC. For instance, Wen et al. found that low levels of serum exosomal miR-29a correlate with a higher recurrence risk in PTC, suggesting its dual utility in diagnosis and prognosis (Wen et al., 2021). Similarly, Dai et al. and Li et al. have highlighted the diagnostic relevance of miR-485-3p and miR-519e-5p, respectively, with the former aiding in risk stratification among PTC patients (Dai D. et al., 2020; Li G. et al., 2023). Further differentiation between PTC and FTC has been achieved through miRNA profiling, revealing distinct patterns of miR-21-5p, miR-221-3p, and miR-181a, achieving 100% sensitivity and 77% specificity (Samsonov et al., 2016). Boufraqech et al.'s work suggests that decreased exosomal miR-145 could serve as a diagnostic tool for cases with inconclusive FNABs (Boufraqech et al., 2014). More precisely, enhanced diagnostic accuracy is reported with miRNA panels, such as those identified by Liang et al., which differentiate PTC from benign thyroid nodules. Liang et al. identified a panel comprising miR-16-2-3p, miR-223-5p, miR-101-3p, and miR-34c-5p, which proves valuable in distinguishing PTC from thyroid nodules (Liang et al., 2020). Meanwhile, Pan et al. suggested the amalgamation of miR-5189-3p and miR-5010-3p within plasma exosomes as prospective biomarkers for diagnosing thyroid nodules (Pan et al., 2020). Additionally, two other miRNAs enriched in plasma exosomes, miR-146b-5p and miR-21a-5p, exhibited notable differential abundance between patients with PTC and those with benign conditions (Delcorte et al., 2022a). Correspondingly, Wang and colleagues identified the upregulation of miR-346, miR-10a-5p, and miR-34a-5p in PTC plasma exosomes (Wang Z. et al., 2019). These findings underscore the significant clinical potential of miRNA panel for TC diagnosis. For LNM, exosomal liquid biopsy is also advantageous for safety, early diagnosis, and dynamic monitoring. Jiang’s study, for example, demonstrated the potential of exosomal miR-146b-5p and miR-222-3p in predicting LNM in PTC (Jiang K. et al., 2020), while other studies have identified novel miRNAs like miR-6774-3p and miR-6879-5p as potential LNM biomarkers in PTC (Chen W. et al., 2022).

### 5.2 Exosomal circRNAs or lncRNAs

Circular RNAs (circRNAs), a subclass of non-coding RNAs with a closed loop structure, are protected within exosomes and have also been identified as promising biomarkers for thyroid cancer diagnosis (Yao and Zhang, 2022). Peng et al. demonstrated that circTACC2 and circBIRC6 were significantly elevated in the serum exosomes of patients with PTC accompanied by LNM (Peng et al., 2024). Similarly, Yang et al. observed altered expressions of circRNAs in serum exosomes from PTC patients, highlighting three specific circRNAs (circ-007293, circ-031752, and circ-020135), with unique patterns that could distinguish PTC cases (Yang et al., 2019b). Additionally, the levels of hsa_circ_0082002 and hsa_circ_0003863 were found to be elevated in PTC patients, correlating positively with clinical indicators such as LNM and vascular invasion, suggesting their potential to assess disease severity (Dai et al., 2023).

Beyond circRNAs, exosomal long non-coding RNAs (lncRNAs) are crucial in TC intercellular communication. Dai et al. reported the upregulation of lncRNA DOCK9-AS2 in PTC, with significant increases found in patients’ plasma exosomes, indicating its relevance in PTC pathology (Dai W. et al., 2020). Furthermore, Liu et al. suggested that elevated levels of lncRNA FGD5-AS1 in blood-derived exosomes might influence TC prognosis and serve as a new diagnostic reference (Liu et al., 2022).

### 5.3 Exosomal proteins

Exosomal proteins have also shown promise in diagnosing TC. A study by Bavisotto et al. revealed that circulating exosomal proteins such as Hsp27, Hsp60, and Hsp90 can effectively distinguish PTC from benign goiter, underscoring their potential as diagnostic biomarkers (Caruso Bavisotto et al., 2019). Additionally, analyses identified activated integrin signaling-related proteins such as TLN1, ITGB2, SRC, and CAPNS1 in serum-purified exosomes from PTC patients with LNM (Luo et al., 2018). In pediatric TC, elevated levels of PD-1 and PD-L1 on exosomes have been associated with clinical outcomes, indicating their diagnostic value (Wang G. et al., 2020). Interestingly, beyond the existence of exosomal proteins in the bloodstream, urinary exosomal thyroglobulin has emerged as a potential early prognostic biomarker for individuals afflicted with PTC (Huang et al., 2020). This innovative finding led to the publication of a patent in Japan in 2017, with further validation from an ongoing study recruiting a larger cohort. Moreover, Wang et al. also reported that urinary exosomal TIMP and Angiotensin-1 are significant markers linked to LNM, corroborated by pathological reports (Wang C. Y. et al., 2022).

These investigations underscore the growing utility of exosomal proteins in TC diagnostics. With advancements in nanotechnologies and single extracellular vesicle analysis, there is optimism that these methods will enhance the identification and utilization of dynamic biomarkers with high accuracy and sensitivity for TC (Ferguson et al., 2022).

## 6 Exosomes as an approach for TC treatment

Recent evidence underscores the significant role of exosomes in the development and progression of TC, highlighting the urgency of translating this knowledge into clinical applications (Kamerkar et al., 2017). Thyroid cancer-derived exosomes, marked by unique biomarkers, are used for detecting cancer and monitoring therapy. Targeting these exosomes to curb disease progression is a key therapeutic strategy. Additionally, exploiting their ability to deliver specific cargoes to targeted cells makes exosomes promising vehicles for precision drug delivery or gene therapy. Given the substantial evidence supporting the role of exosomes in the TC tumor microenvironment, they also hold potential as adjunctive agents in radiotherapy and immunotherapy for TC (Table 3).

**TABLE 3:** Exosome-mediated therapies in thyroid cancer.

	Approaches	Effect	Reference
Therapeutic target	Exosomal miR-152	Inhibit TC cell proliferation, migration and invasion by binding with DPP-4	Tang et al. (2020)
	Silencing miRNA423-5p in exosomes	Inhibit PTC cell migration and invasion	Ye et al. (2019)
Drug delivery	Exosomes loaded SCD-1 siRNA	Inhibit ATC cellular proliferation and promote cellular apoptosis	Wang M. H. et al. (2022)
	Combining irradiation and chemotherapy by means of iRGD-targeted exosomes	Delivering ^131^I and doxorubicin to ATC cells, leading to significant tumor growth inhibition in mouse model	Wang C. et al. (2022)
	Exosomes from the cultured ATC cells	Exosomes were targeted into ATC cells	Gangadaran et al. (2018)
Radiotherapy Immunotherapy	Exosomes used as vehicles for TKIs	Re-establishing radioiodine-sensitivity	Rajendran et al. (2021)
	NK-cell-derived exosomes treated with IL-15	Showed higher cytolytic activity toward human thyroid cancer cells	Zhu et al. (2019)
	NK-cell-derived exosomes	Exert strong killing effects to ATC cells	Zhu et al. (2018)

DPP4, Dipeptidyl dipeptidase 4; TKIs, Tyrosine kinase inhibitors; IL-15, Interleukin-15; NK, Nature killing cell.

### 6.1 Exosome as therapeutic target

As previously discussed, exosomes play a pivotal role in orchestrating interactions between TC cells and non-cancer cells, thereby facilitating the progression of TC. Consequently, the strategic targeting of exosome-mediated tumor-initiated intercellular communication has emerged as an appealing avenue for TC therapy. Presently, exosome-targeted therapeutic strategies can be categorized into three principal domains: modulation of exosome function, attenuation of tumor exosome secretion, and interference with the uptake of tumor-exosomes by recipient cells (Wang, 2022).

Initially, direct blockade of exosome cargos, or inhibition of exosome-mediated cell-cell communication, is expected to be more effective when combined with surgical resection or cytotoxic therapies. In a study by Tang et al., it was revealed that exosomal miR-152 possesses the ability to impede the proliferation, migration, and invasion of TC cells through its interaction with Dipeptidyl dipeptidase 4, thus presenting a potential novel target for TC treatment (Tang et al., 2020). Likewise, Ye et al. conducted functional experiments showcasing that the overexpression of miRNA423-5p within exosomes enhances PTC cell migration and invasion, while the silencing of miRNA423-5p in exosomes elicits the converse impact (Ye et al., 2019). These findings suggest that targeted suppression of specific exosomal components could represent a promising therapeutic strategy for combating PTC.

Beyond the direct suppression of exosomes, various chemical compounds have demonstrated the capability to impede exosome secretion. For example, GW4869, a recognized inhibitor of exosome secretion, is frequently used *in vivo* to reduce exosome release. In a colorectal cancer model, exosomes derived from cancer cells were shown to promote tumor growth by enhancing cell proliferation and inhibiting apoptosis. Conversely, intra-tumoral administration of GW4869 was found to suppress tumor growth (Wang B. et al., 2019). This approach further offers an alternative avenue for exosome-mediated TC therapy. Nevertheless, in the context of future TC treatments, the potential off-target effects of these chemical substances necessitate elucidation. Furthermore, a deeper understanding is imperative to determine the optimal threshold influencing exosome secretion.

In conjunction with these strategies, efforts have been focused on inhibiting the cellular uptake of tumor-derived exosomes to block their impact on target cells (Wang, 2022). A notable example is the use of heparin to impede exosome uptake in a breast cancer mouse model (Wills et al., 2021). Due to its natural origin, heparin possesses an excellent safety profile, making it a promising therapeutic candidate for obstructing the uptake of tumor-derived exosomes. As a result, heparin and its derivatives offer potential avenues for future TC treatment.

### 6.2 Exosome-mediated drug delivery

Exosomes surpass other lipidic vesicles as superior nanocarriers owing to their enhanced targeting capacity (El Andaloussi et al., 2013) and heightened binding affinity (Kibria et al., 2018). Notably, exosomes derived from cancer cells exhibit specific tropism that can be harnessed for drug delivery into tumor cells (Feng et al., 2020). Therefore, the encapsulation of anticancer drugs within exosomes holds substantial promise for tumor-targeted therapy. In a recent study, exosomes loaded with triptolide exhibited notable encapsulation efficiency and demonstrated the ability to selectively induce apoptosis in ovarian cancer cells (Liu et al., 2019). Srivastava and colleagues additionally formulated and assessed doxorubicin-loaded exosomes for the treatment of lung cancer. The investigators concluded that these exosomes effectively reached the cancer cell niche, inducing a cytotoxic effect specifically on cancer cells (Srivastava et al., 2016). Regarding the impact of drug-loaded exosomes on TC treatment, Gangadaran and co-researchers isolated exosomes from cultured ATC cells to assess the targeting competence of tumor-derived exosomes towards their parental tumor within a mouse model. The exosomes administered to ATC model mice were promptly internalized by ATC tumors, accumulating within the tumor region within 30 min (Gangadaran et al., 2018). Employing this approach, Melzer and co-authors prompted the development of profoundly metastatic breast cancer tumors in NODscid mice. Notably, intravenous administration of taxol-loaded exosomes yielded a notable 60% decrease in subcutaneous tumors alongside a 50% reduction in the count of distant metastases (Melzer et al., 2019). Although these outcomes mirrored those of the control group subjected solely to taxol application, it is noteworthy that the concentration of taxol within exosomes was remarkably lower by a factor of 1,000. These findings suggest that exosomes hold the potential to serve as a viable alternative for cancer treatment, offering the advantage of minimal side effects. In the realm of exosome-mediated drug delivery for TC therapy, Wang et al. innovatively devised a dual antitumor approach that amalgamates internal irradiation and chemotherapy. This strategy employs iRGD-targeted exosomes as delivery vectors, facilitating the transport of Iodine-131 and doxorubicin to ATC cells (Wang C. et al., 2022). Beyond drug cargos, a separate investigation demonstrated that exosomes loaded with SCD-1 siRNA effectively curbed ATC cellular proliferation. The underlying mechanisms potentially involve the modulation of fatty acid metabolism and regulation of ROS levels (Wang C. et al., 2022). These findings suggest that exosomes, whether derived from natural tumor cells or engineered counterparts, could serve as highly effective carriers for future TC therapeutic drug delivery.

### 6.3 Exosome-mediated radiotherapy and immunotherapy

Radioactive iodine represents an alternative therapeutic avenue for TC, utilizing radiation to impede TC progression and induce apoptosis in cancer cells. Nonetheless, the tumor microenvironment, marked by specific conditions like hypoxia, can foster radioresistance in cancer cells. Recent investigations have focused on elucidating the involvement of exosomes in conferring radioresistance upon cancer cells. Notably, an experimental study has illuminated the capacity of cancer-associated fibroblasts to release exosomes that foster stemness in colorectal cancer cells, thereby provoking radioresistance (Liu et al., 2020). In another investigation, Rajendran and colleagues proposed a novel utilization of exosomes derived from primary human adipose-derived stem cells. These exosomes have been repurposed as delivery vectors for tyrosine kinase inhibitors due to their capacity for efficient loading and uptake by TC cells refractory to radioactive iodine treatment (Rajendran et al., 2021). In conjunction with these findings, it is plausible to surmise that compounds specifically targeted to exosomes may constitute a viable strategy for addressing refractory Iodine-131 TC therapy.

Exosomes have also been engineered for use in cancer immunotherapies due to their ability to support an immunomodulatory microenvironment. Engineered exosomes, loaded with bioactive agents, are designed to activate various stages of the cancer immunity cycle, culminating in robust cancer-specific immune responses (Nam et al., 2020). Moreover, bioengineered exosomes show enhanced stability in circulation, as they are less susceptible to macrophage-mediated phagocytosis, thereby increasing their biostability. In a separate investigation, Plebanek et al. demonstrated that exosomes from poorly metastatic melanoma cells promote immune surveillance by mobilizing patrolling monocytes, leading to the elimination of cancer cells within the pre-metastatic niche (Plebanek et al., 2017). In the context of exosome-mediated TC immunotherapies, Zhu and collaborators illustrated that exosome mimetics originating from NK cells manifest robust cytotoxic effects against cancer cells, encompassing ATC cells (Zhu et al., 2018). This research group delved deeper into the prospects of NK-cell-derived exosomes for immunotherapy by pre-conditioning with IL-15. The resultant exosomes, subjected to IL-15 treatment, displayed markedly elevated cytolytic activity directed at human thyroid cancer cells. Furthermore, this treatment led to a concomitant augmentation in the expression of molecules linked to NK-cell-mediated cytotoxicity (Zhu et al., 2019).

Collectively, owing to the protective function of their bilayer membranes, exosomes excel as promising drug carriers. Their robust biocompatibility and precise targeting capabilities position them as a promising new approach for treating TC. However, the clinical implementation of exosome-based therapies remains a future prospect, necessitating extensive experimental investigation into additional biomarkers and exosomal targets. Simultaneously, recent advances in engineered exosomes have unveiled innovative avenues for exosome-driven therapies in TC.

## 7 Conclusion and outlooks

This review provides a comprehensive summary of recent discoveries and advancements regarding the involvement of exosomes in the development and diagnosis of TC. Furthermore, we underscore the significant potential of exosomes across various clinical applications, broadening the scope for innovative TC therapies. Unlike traditional approaches, exosomes offer a minimally invasive option for both diagnosis and continuous monitoring of TC, marking a new era in precision oncology. Future research is anticipated to confirm the therapeutic efficacy of exosome-based interventions, potentially extending their benefits to a broader spectrum of TC patients.

Despite significant advancements in exosome research, several challenges persist in translating these findings into clinical practice. One pressing challenge is detecting TC-derived exosomes in patients with small to medium-sized tumors, which requires further large-scale studies to thoroughly assess the accuracy and sensitivity of exosomal biomarkers (Delcorte et al., 2022b). Additionally, the implications of tumor-derived exosomes as therapeutic agents need careful consideration due to their potential dual effects on tumor progression. Optimizing exosome isolation techniques is critical to ensure cost-effective, large-scale production that could facilitate their integration into clinical settings (Grangier et al., 2021). Continued research into exosomes will deepen our understanding of their multifaceted roles in thyroid cancer and support the development of innovative diagnostic and therapeutic strategies. With advancements in nanotechnology and bioengineering, exosomes are poised to revolutionize the approach to thyroid cancer management, promising significant improvements in diagnostic accuracy and treatment efficacy.
